# Polymer Mixtures for Experimental Self-Limited Dental Burs Development—A Preliminary Approach (Part 1)

**DOI:** 10.3390/jfb14090447

**Published:** 2023-08-29

**Authors:** Radu Marcel Chisnoiu, Alexandrina Muntean, Ovidiu Păstrav, Andrea Maria Chisnoiu, Stanca Cuc, Laura Silaghi Dumitrescu, Mihaela Păstrav, Doina Prodan, Ada Gabriela Delean

**Affiliations:** 1 Department of Odontology, Endodontics and Oral Pathology, “Iuliu Hațieganu” University of Medicine and Pharmacy, 33 Moților Street, 400001 Cluj-Napoca, Romania; marcel.chisnoiu@umfcluj.ro (R.M.C.); ovidiu.pastrav@umfcluj.ro (O.P.); ada.delean@umfcluj.ro (A.G.D.); 2 Department of Pedodontics, “Iuliu Hațieganu” University of Medicine and Pharmacy, 31 Avram Iancu Street, 400083 Cluj-Napoca, Romania; alexandrina.muntean@umfcluj.ro; 3 Department of Prosthodontics, “Iuliu Hațieganu” University of Medicine and Pharmacy, 32 Clinicilor Street, 400006 Cluj-Napoca, Romania; 4 “Raluca Ripan” Institute for Research in Chemistry, Babes-Bolyai University, 30 Fantanele Street, 400294 Cluj-Napoca, Romania; laura.silaghi@ubbcluj.ro (L.S.D.); doina.prodan@ubbcluj.ro (D.P.); 5 Department of Orthodontics, “Iuliu Hațieganu” University of Medicine and Pharmacy, 31 Avram Iancu Street, 400083 Cluj-Napoca, Romania; mihaela.pastrav@umfcluj.ro

**Keywords:** polymer mixture, graphene, mechanical properties, dentistry, dental materials

## Abstract

Alternative techniques have been investigated for effectiveness in caries removal because conventional metallic dental burs can lead to an excessive loss of sound tissue. The aim of the present study is to realize a preliminary approach in obtaining effective polymer mixtures for polymeric bur development, capable of removing primary dental caries using combinations of polymers to ensure the requirements for such instruments, but also a greater compatibility with the teeth structure. This study assessed the main mechanical properties, water sorption, solubility and microscopic structure of four new polymer mixture recipes to provide essential features in obtaining experimental self-limited dental burs. Two mixtures have in their composition polymer mixtures of Bis-phenol A diglycidyl ether dimethacrylate/Triethylene glycol dimethacrylate/Urethane dimethacrylates (R1, R2), and two other mixtures have Bis-phenol A diglycidyl ether dimethacrylate/Polymethyl methacrylate/Methyl methacrylates (R3, R4). The incorporation of nanoparticles into the polymer matrix has become essential due to the need of polymer biocompatibility increasing along with teeth surface remineralization, so that the powder charge was added to four recipes, such as 5% glass with BaF_2_ and 0.5% graphene with silver particles. All data sets were analyzed using the One-Way ANOVA test. R3, R4 showed higher compressive strength and diametrical compression values; these values increased when glass and graphene were added. Moreover, the addition of glass particles lead to an increase in flexural strength. Regarding the sorption, sample R3 had the most significant differences between day 69 and the rest of the investigation days, while the solubility varied at different intervals. From the mechanical evaluation, we could conclude that the Bis-GMA/PMMA/MMA mixtures fit the mechanical characteristics supported by polymer burs, following future studies regarding their use on the affected dentin.

## 1. Introduction

Dental periodic check-ups have become common for the modern man, for whom health is a priority. Following these periodic visits, dental caries have been discovered in their early stages, so that the intervention to remove them is easier and less painful. The new trends in current practice is to maintain the tooth surface as much as possible, as well as its structure.

In the last century, there was no substantial development of the instruments used for the preparation of dental cavities, which is similar to that used many generations ago. Even though, at present, there are dental burs of various shapes and sizes, as well as different manufacturing materials—stainless steel, tungsten carbide, and diamond burs—and they operate based on the same principle—applied in contact with tooth surfaces, using rotating movements, and indiscriminately removing hard and soft dental tissues.

Because conventional carbide or steel burs can lead to an excessive loss of sound tissue, alternative techniques have been investigated for effectiveness in caries removal [1]. This is how polymer burs appeared, with the concept of minimally invasive dentistry, showing their self-limiting capacity [2]. These burs are made of polymers that must meet the conditions of being harder than soft dentin, but softer than sound, healthy dentin, with the principle being based on selectivity [3]. These burs have been shown to cut less dentinal tubules; therefore, less pain sensations are triggered compared to conventional bur usage [4].

Bond strengths were also tested in the remaining dentin walls after using polymeric burs instead of conventional ones, demonstrating the low bond strength when the first ones are used [5]. Due to the formation of a debris layer by polymer bur-wearing, the penetration ability of the bonding agent is affected [6,7].

The literature that refers to these polymeric burs is strictly related to their effectiveness in caries removal, without any studies regarding the type of composition and their characteristics [8]. When their composition is mentioned, only a medical poly-ether-ketone-ketone is specified [9].

Due to these advantages and the few studies reported on the types of polymers used in bur manufacturing, the aim of this study was to obtain effective polymer mixtures for polymeric burs’ development, in order to remove primary dental caries using combinations of polymers to ensure the requirements for such instruments, but also having a greater compatibility with the teeth structure and restorative material bonding agents. This represents a preliminary approach for a new material designated for polymeric dental burs’ manufacturing [1,10].

We attempted to obtain some polymer mixtures using common components used in dental materials’ composition for direct or indirect restoration: Bis-phenol A diglycidyl ether dimethacrylate (Bis-GMA), and Polymethyl methacrylate (PMMA), which was used for prosthetic dental applications, including the manufacture of artificial teeth or dental prostheses and dental obturation materials. These polymers offer the desired resistance to the mixtures, and, also, offer good biocompatibility [11]. For good handling and flexibility, the base polymers are mixed with diluting monomers such as triethylene glycol dimethacrylate (TEGDMA), urethane dimethacrylate (UDMA) or methyl methacrylates (MMA) [12].

The incorporation of nanoparticles into the polymer matrix has become essential due to the need to improve the properties of the polymer in terms of the biocompatibility and remineralization of teeth [13]. Graphene has shown interesting results in many applications, emerging as a promising material for incorporation into polymer matrices [14]. Its high specific surface area and lightness, as well as mechanical, thermal and electrical properties, allow for the production of high-performance nanocomposites. Furthermore, the functionalization of graphene particles with elements that are added to the final product is a major advantage for its use in the composition of polymer mixtures [15]. For example, the addition of glass with BaF_2_ has an anticariogenic potential in polymers’ matrix [16], and Ag particles have an antibacterial effect in all dental applications [17].

The performance of dental composites depends on many factors, such as the structures and ratios of monomers and the type of powder used [18,19]. Therefore, after they are obtained, they are subjected to characterization tests and the measurement of the various parameters in order to be included in the targeted material category.

## 2. Materials and Methods

### 2.1. Dental Materials’ Preparation

Eight recipes of polymer mixtures with different ratios and compositions were obtained according to Table 1. Two types of Bis-GMA were used: a commercially available one, Bis-GMAimp (Sigma-Aldrich, Merck, Kenilworth, NJ, USA), and Bis-GMA (2018), manufactured at “Raluca Ripan” Institute for Research in Chemistry, Cluj-Napoca, Romania [20,21]. All other organic substances and initiation system were used from Sigma-Aldrich, Darmstadt, Germany.

The inorganic phase was synthesized in our laboratory (UBB-ICCRR, Babes-Bolyai University, Raluca Ripan Institute of Research in Chemistry). Glass filler with BaF_2_ (size 2–6 nm) was obtained by the conventional melting method, and the graphene containing 0.5% Ag was obtained by Hummer’s method from graphite [22].

### 2.2. Characterization

#### 2.2.1. Scanning Electron Microscopy (SEM)

Scanning electron microscopy (SEM) images of the samples were obtained using an electron microscope FEI company (Hillsboro, OR, USA). The samples used in the diametrical compression mechanical resistance tests were initially investigated under vacuum at a magnification of 1000 and 30 kV.

#### 2.2.2. Mechanical Resistance Tests

The Flexural Strength

The flexural strength of the materials was measured according to the 3-point bending method presented in ISO 4049 [23] for dental materials. Rectangular Teflon molds of 2 mm × 2  mm × 25 mm were used to prepare uniform bars. The samples were distributed in rectangular shapes and photopolymerized with a dental LED light curing lamp (Woodpecker Medical Instrument Co., Guilin, China), 850 W, in 5 points for 20 s/point, to obtain solid specimens. A total of 10 samples were prepared for each tested recipe. The polymerized samples were placed in tubes with artificial saliva at 37 °C for 24 h before measuring the flexural strength and the Young modulus, in order to describe the ability to tolerate compression or elongation using the Lloyd LR5k Plus dual-column mechanical testing machine (Ametek/Lloyd Instruments, Meerbusch, Germany).

Compression Resistance

Teflon molds with a height of 6 mm and an inner diameter of 3 mm were used to prepare the uniform samples. The samples were photopolymerized with a dental LED light curing lamp both on the upper and lower sides of the mold for 20 s on each side, and then on the thickness of the samples for another 40 s. All samples (*n* = 10) were tested vertically on a computer-controlled Lloyd LR5k Plus dual-column mechanical testing machine and tested with a 5 kN load cell at a transverse speed of 1 mm/min.

Resistance to Diametrical Compression

The diametral compression was measured using specimens prepared in Teflon molds with dimensions of 3 mm in height and 6 mm in diameter. The samples were mechanically tested on their diameter by subjecting them to a force of 0.5 mN/s using the mechanical testing machine. The NexygenPLUS software was used (version 4.1, Ametek GmbH, Meerbusch, Germany) to calculate and express the diametral stress resistance from the stress/force curve. An average of 10 trials was reported for each recipe.

#### 2.2.3. Water Sorption (*WS*) and Solubility (*SL*) Tests

Water sorption and solubility were determined according to ISO 4049 [23] specifications, with an extended immersion time of 69 days. Teflon molds with a diameter of 20 mm and a thickness of 2 mm were used to obtain uniform discs. The materials were evenly placed in molds and photopolymerized in 5 points using LED light-curing lamp on both sides to produce solid discs. A total of 10 specimens were prepared for each sample. After polymerization, the samples were placed in a desiccator for 24 h, and, then, each sample was weighed until a constant mass (*m*1) was obtained, using an analytical balance (Adventurer^®^ Analytical, Ohaus AX224M, OHAUS Corporation, Parsippany-Troy Hills, NJ, USA). Five of each sample were placed in an individual container and immersed in 10 mL of simulated body fluid (SBF) (Na_2_HPO_4_, KH_2_PO_4_, H_2_O) and were placed five in artificial saliva (Na_2_HPO_4_, NaHCO_3_, CaCl_2_, HCl-1M, H_2_O) and stored in a water bath at 37 °C. At different time intervals (1, 2, 5, 6, 7, 14, 24 and 69 days), the samples were removed from the immersion medium, wiped with absorbent paper and weighed (*m*2). After weighing, they were stored in a desiccator for 2 h and weighed again (*m*3), and returned to containers with 2 mL of freshly distilled water. The water sorption (*WS*) and solubility (*SL*) of the samples were determined using the following formulas:

(1) $$WS=\frac{m2-m1}{m1}\times 100$$

(2) $$SL=\frac{m1-m3}{m1}\times 100\;$$

#### 2.2.4. Determination of Abrasion Resistance

The general technique for the preparation and testing of the specimens was similar to that described for the determination of the compressive strength, with cylindrical specimens having dimensions of 6 mm × 3 mm (height × diameter). The sample thus obtained was immersed in distilled water at 37 ± 1 °C, where it was kept for 24 h. After 24 h, the specimen was removed and dried, and the cross-sectional dimensions were measured with a micrometer and weighed on an analytical balance (Adventurer^®^ Analytical, Ohaus AX224M, OHAUS Corporation, Parsippany-Troy Hills, NJ, USA) before and after abrasion. We made 10 samples of each recipe (*n* = 10). Abrasion resistance was achieved with the KAVO 11 micromotor (Kavo, Biberach, Germany). The standard abrasion interval was set at 300,000 revolutions/minute for 30 s of grinding. The samples were eroded on Optidisc-Kerr discs (Kerr Dental, Kloten, Switzerland), 15 Art. No. 198, with polishing with large, medium and small grain polypants. The disks and rubbers used were attached to the contra-angle piece, applying circular movements. The evaluation of the samples was conducted by measuring the height of the samples and weighing them, both before and after processing in order to observe the losses of the substance.

#### 2.2.5. Statistical Analyses

For each group of specimens, the results were subjected to a statistical analysis for the calculation of median and standard deviations. All data sets were analyzed using the One-Way ANOVA test. Comparisons between the materials were performed using Tukey’s test. The statistical significance was set at *p* < 0.05. The statistical analysis was performed using the Origin2019b Graphing & Analysis program (version 2019b, OriginLab Co., Northampton, MA, USA).

## 3. Results

### 3.1. Scanning Electron Microscopy (SEM)

Samples’ morphology and filler distribution into the formed composites were investigated by SEM microscopy.

**Figure 1:**
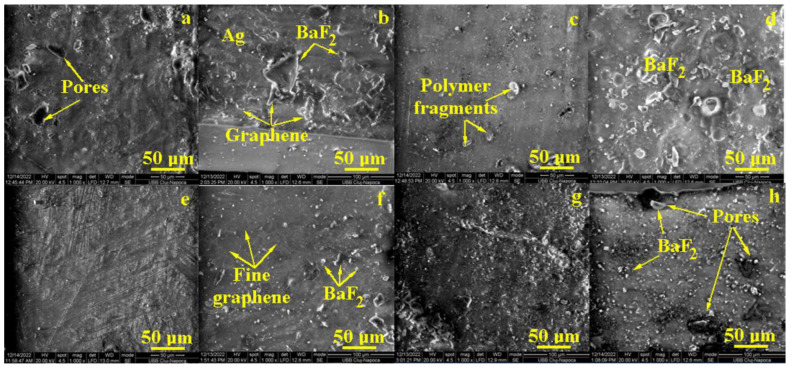
SEM images of the samples after photo polymerization: (**a**) R1, (**b**) R1 + P, (**c**) R2, (**d**) R2 + P, (**e**) R3, (**f**) R3 + P, (**g**) R4 and (**h**) R4 + P.

The R1 sample features a homogeneous mixture of Bis-GMAimp 40%, UDMA 20% and TEGDMA 40% into a compact matrix, where some superficial pores occur with elongated shapes and sizes ranging from 15 to 25 μm (Figure 1a). The filler addition of R1 + P samples, respectively, reveals a merely uniform distribution of bigger BaF_2_ glass clusters with polyhedral shapes ranging from 10 to 50 μm. Some graphene folds were observed when the nanostructural mixture was embedded into the polymer matrix (Figure 1b). The upper right corner of Figure 1b reveals an area where Ag nanoparticles are very finely mixed up with graphene oxide.

The R2 composition based on Bis-GMA (2018) presents a more uniform and compact polymeric matrix without pores but with several segments of polymer from the surface of the sample, detached during extraction from the mold (Figure 1c). The filler addition, sample R2 + P, conducts to an interesting microstructure with bigger BaF_2_ particles uniformly distributed in the compact matrix (Figure 1d). They present merely rounded shapes due to the good lamination of the particle corners, and their sizes vary from 5 to 25 μm. The nanostructural components such as Ag and graphene are very well dispersed into the polymer that are not distinguished among the other microstructural features already discussed.

The R3 sample contains Bis-GMA imp 10%; UDMA 25%; PMMA 40%; MMA 25% that were very well mixed during the polymerization process, forming a coherent and compact matrix (Figure 1e). Some superficial irregularities are observed but without flaws or pores. The R3 + P sample (Figure 1f) shows an uniform distribution of BaF_2_ particles ranging from 3 to 10 μm. The nanostructural features are almost invisible due to their optimal mixing into the polymer matrix, but some graphene lines are still observed in the upper left side (Figure 1f).

The R4 sample presents a corrugated surface with a lot of irregularities that might occur due to the imperfect contact of the upper side of the mold, but the matrix bulk looks compact and cohesive (Figure 1g). The filler addition in sample R4 + P (Figure 1h) induces the presence of significant pores associated with bigger BaF_2_ particles (e.g., sizes ranging from 10 to 15 μm). The areas containing small filler particles are uniform and prove a good cohesion of the composite. However, the pores’ presence might affect the mechanical properties and/or the liquid absorption.

### 3.2. Mechanical Properties

The results of the mechanical properties are influenced by the composition of each recipe.

**Figure 2:**
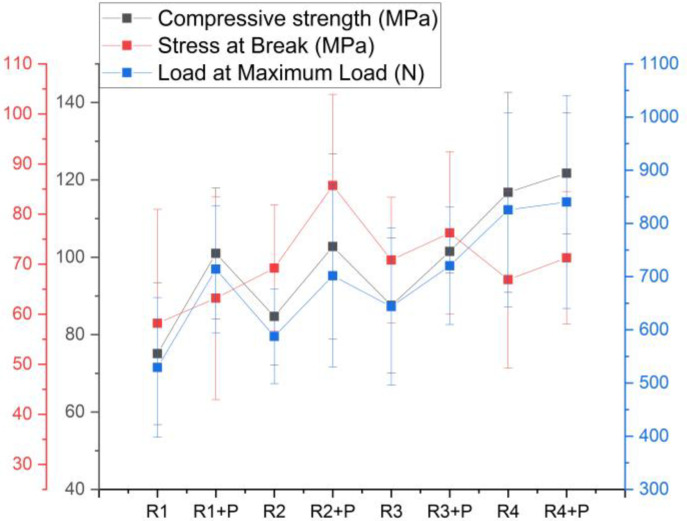
Compression test parameters for the investigated mixtures.

Figure 2 shows the relationship between the compressive strength and composition of polymers’ mixture. It can be observed that the strength of the Bis-GMA/UDMA/TEGDMA polymers is lower than the Bis-GMA/PMMA/MMA mixture, by up to 10%. If the compressive strength is compared according to the added powder, it can be observed that the strength increases when glass and graphene are added.

The highest stress sustained until the specimen yielded or the first crack appeared is sample R2 + P, and the maximum force sustained of 840 N was for sample R4 + P. From the three characteristics obtained from the deformation curve of the samples, it can be observed that R1 is the most fragile material, and the most curved curve is that of sample R4 + P.

Statistical analysis results after the Anova one way test obtained a value of *p* < 0.05; thus, there are differences between the eight investigated recipes. The Tukey test obtained the differences between R1 and R4, and there are no differences between the samples with and without powder.

**Figure 3:**
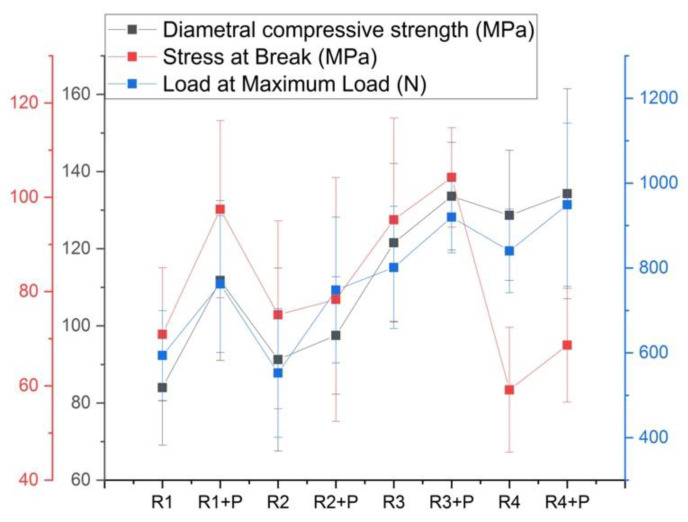
Diametral compression test parameters for the investigated mixtures.

Figure 3 shows the resistance to diametrical compression of the mixtures with the same ratios of different polymers. The samples R3 and R4 showed a higher resistance than the mixtures R1 and R2, and the samples with powder are always better performing than the empty polymer matrix. As in the case of the compression test, the sample that bears the least stress is R4 and R4 + P; however, with a higher load at the maximum load, it therefore is the most ductile experimental material.

The statistical analysis indicated differences between the R3 + P recipe and the rest of the samples, with this one having the highest flexural strength value (*p* < 0.05), as well as the highest load at the maximum load.

**Figure 4:**
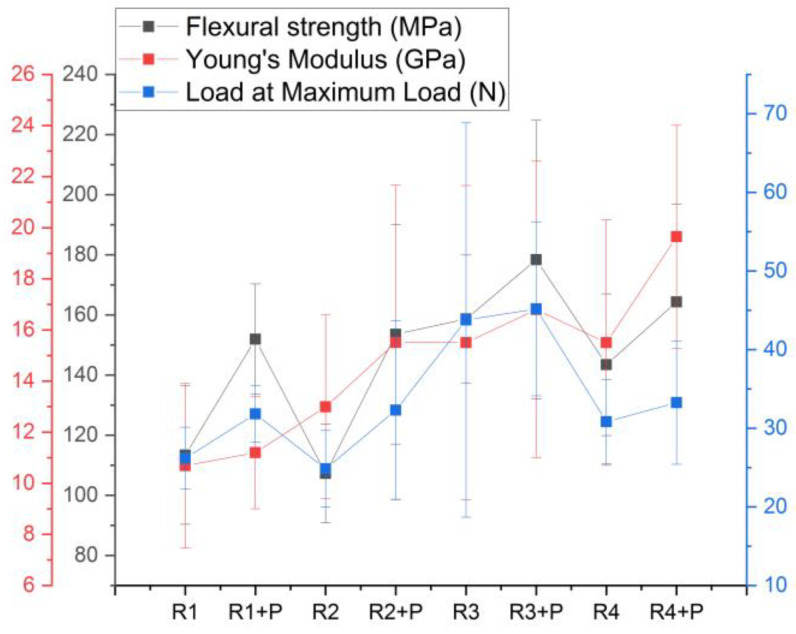
Flexural strength test parameters for the investigated mixtures.

The addition of glass particles into the polymer matrix, regardless of its type, leads to an increase in flexural strength, while for the Young’s modulus of elasticity, the differences are small between the groups with and without powder, showing differences between the investigated groups (*p* < 0.05), and more specifically, between R4 + P and the rest of the samples (Figure 4).

Considering the mechanical properties of the samples with the addition of the inorganic phase, all of them demonstrated better mechanical properties than the pure samples. The greatest increase in the presence of inorganic powders was observed in the flexural strength by up to 50% as compared to pure samples, followed by 30% in the diametral compression test and 20% in the compression strength test.

### 3.3. Water Sorption and Solubility

Depending on the composition of the investigated mixtures, the samples immersed in SBF and artificial saliva for 69 days presented different sorption values. 

**Figure 5:**
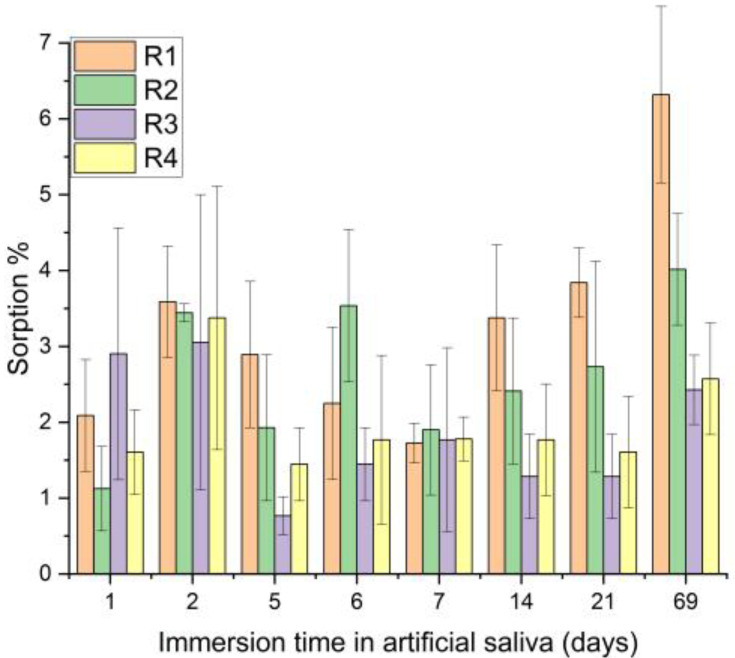

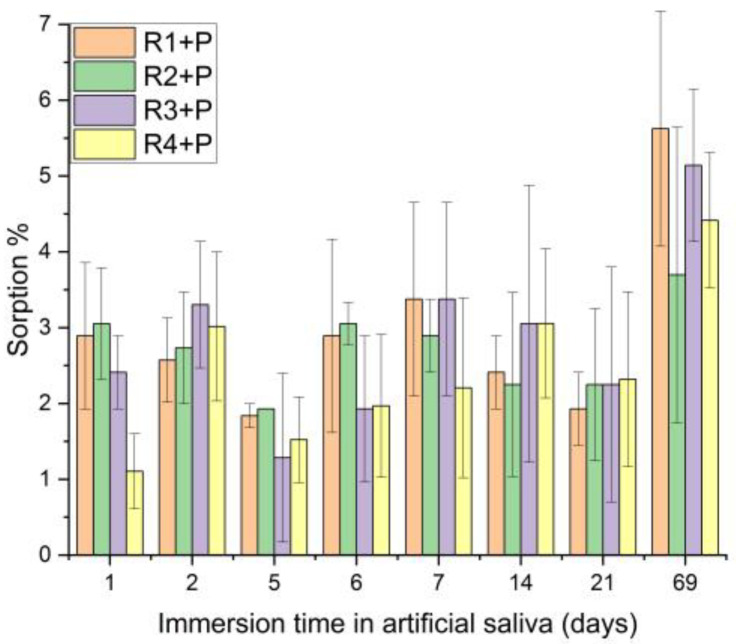
Sorption in artificial saliva, depending on the immersion time.

The three weightings of each sample were subjected to statistical analysis. The results were obtained within each sample investigated for 69 days of immersion in saliva; there were samples that did not show significant differences during the investigated period (R2 + P, R3, R4, R4 + P), as well as samples with small significant differences (R2, R3 + P), but there were also samples with significant differences between all investigated days and the last day, day 69 (R1 + P and R1) (Figure 5).

**Figure 6:**
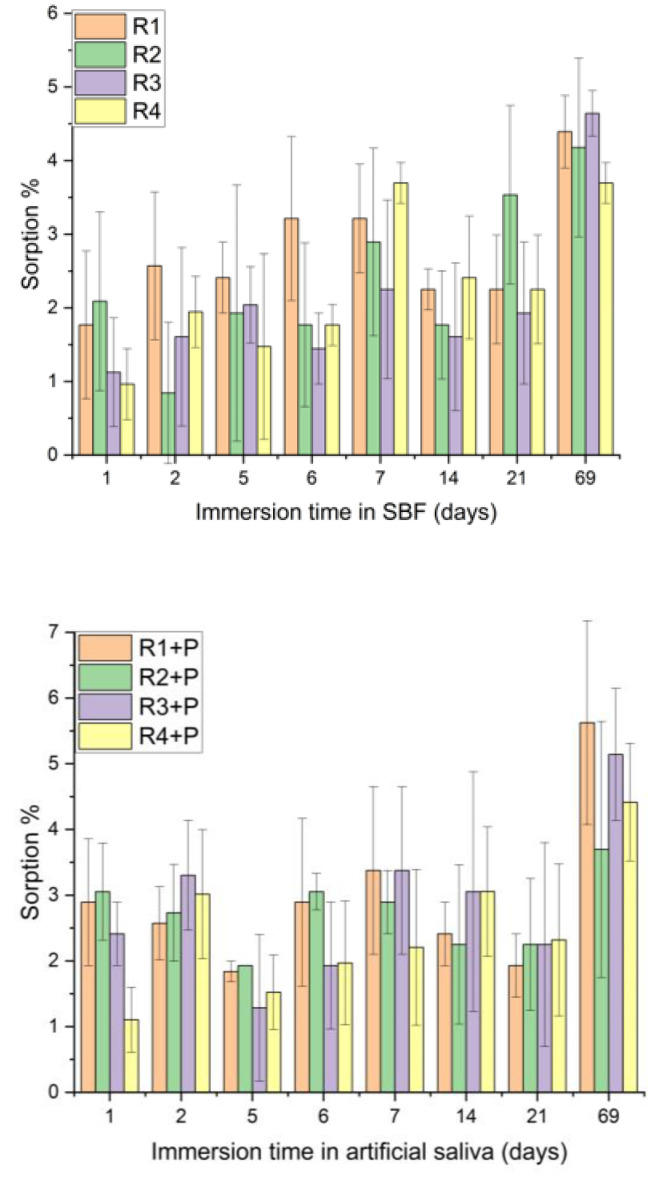
Sorption in SBF, depending on the immersion time.

Considering the statistical analysis for the SBF immersion results, it can be observed that samples R2 and R2 + P do not have any statistically significant differences during the investigation period. Samples R1, R4 and R1 + P and R4 + P have significant differences between day 1 and day 69, while sample R3 has the most significant differences between day 69 and the rest of the investigation days (Figure 6).

Superimposing the two graphs and statistically analyzing the evolution of each sample immersed in saliva and SBF, there are no significant differences for any of the investigated samples, as the p values are higher than the significance level of 0.05.

Comparing the sorption values, there were no significant differences between the samples, neither for those immersed in saliva (*p* = 0.05122) nor for the group immersed in SBF (*p* = 0.69341).

**Figure 7:**
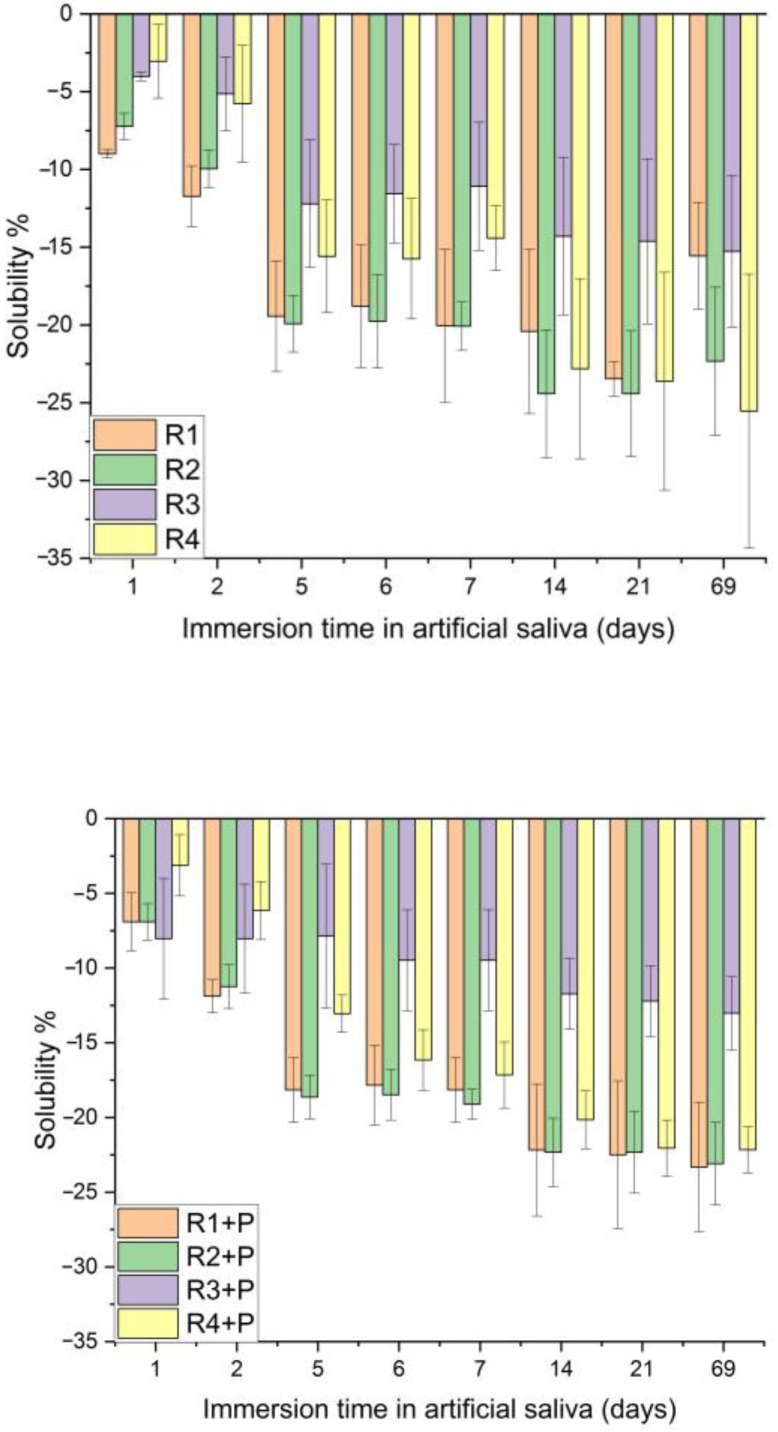
Solubility in artificial saliva, depending on the immersion time.

Regarding the solubility, the statistical analysis pointed out a significant difference for all the samples immersed in saliva, the exception being sample R3 and R3 + P. If, for R1 and R1 + P, the differences are between day 1 and days 7, 14, 21 and 69, for samples R2, R2 + P and R4, the differences are between days 1 and 2 and days from 7 onwards (Figure 7).

**Figure 8:**
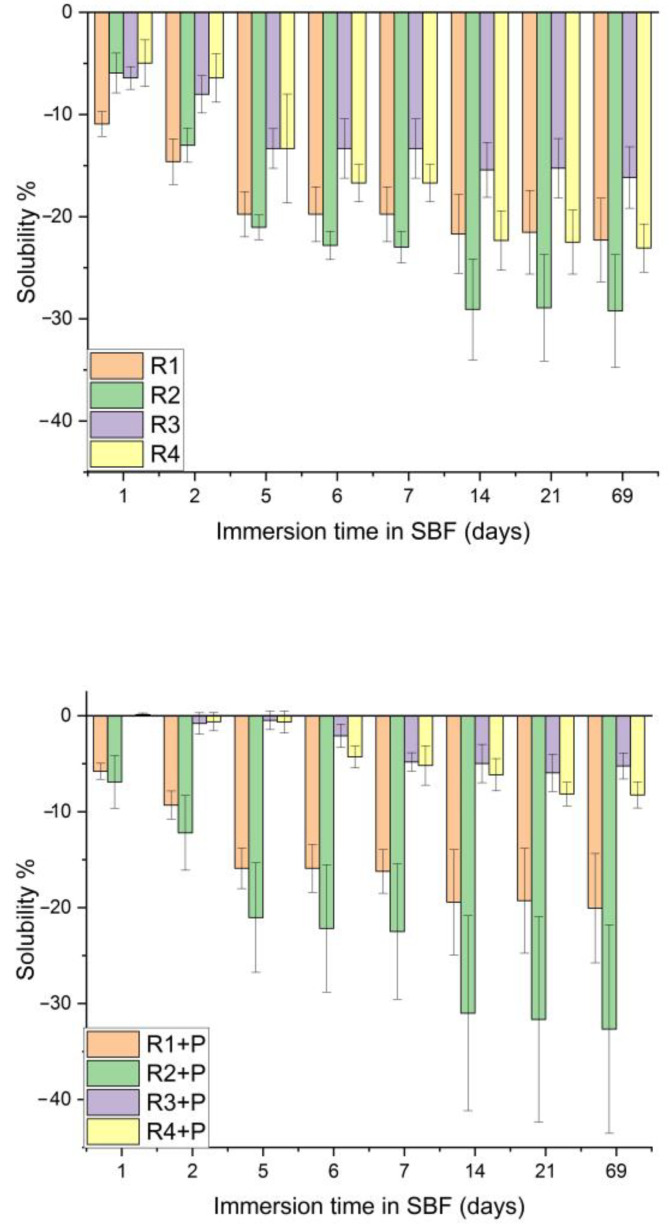
Solubility in SBF, depending on the immersion time.

Analyzing the statistical results, *p* values below the significance level are obtained for each sample investigated within the 69 days of solubility analysis in SBF, a sign that this characteristic of the samples has variations and different behaviors during the analysis period. Following the Tukey test, it can be observed that for R1 and R2, the differences appear between day 1 and days 6, 7, 14, 21 and 69, and for R1 + P and R2 + P, the differences appear between day 1 and day 14, 21 and 69 (Figure 8).

Superimposing the two graphs and statistically analyzing the evolution of each sample immersed in saliva and SBF, there were significant differences between the investigated samples, with the R3 + P sample showing significant differences in the solubility trajectory for 69 days of saliva as compared to SBF.

Comparing the solubility values between the investigated samples, there were significant differences between the samples, both for those immersed in saliva (*p* = 0.0124) and for the group immersed in SBF (*p* < 0.05). Following the Tukey test, differences were noticed between the R3 + P and R4 + P samples and the rest of the investigated samples.

Comparing the evolution of each sample according to the immersion time in different environments, each one presented different p values according to Table 2. R2 + P did not show any difference in the studied interval in the evaluation of sorption, neither in the saliva nor in the SBF, and R4 + P did not show statistically significant differences in the evaluation of the solubility regardless of the immersion medium.

Following this test, it can be concluded that water absorption is directly proportional to the addition of filler powders in the composition, as compositions with PMMA have a lower absorption than those with TEGDMA.

### 3.4. Abrasion Resistance

The obtained values provide useful information on the wear behavior of the material obtained for experimental burs (Table 3). 

Comparing the differences in height and weight after abrasion, it can be observed that the samples with powder had a higher resistance to abrasion than the pure samples. There were no statistically significant differences between the baseline and after cycles in height and weight (*p* > 0.05), with the abrasion resistance increasing from R1 to R4. 

## 4. Discussion

In order to overcome the major inconvenience of metallic burs (the very high hardness), a softer material made from a polymeric resin mixture can be used in order to develop an auto-limited bur which will remove the infected dentin and will blunt out when in contact with the harder, healthy dentin, in order to prevent any unnecessary cutting. Moreover, residual sawdust resulting from bur wear that has an disinfectant effect on remnant dentin is a desired effect; this study represents an approach in obtaining such material.

In the present study, several compositions of mixtures based on Bis-GMA/UDMA/TEGDMA and Bis-GMA/PMMA/MMA monomers were prepared. They were reinforced with fillers to improve the mechanical properties of the mixtures. Currently and in general, several types of monomers are used, for example, Bis-GMA, TEGDMA and UDMA [24], as the basis of different dental materials [25].

The ratio and combinations of polymers were based on the multitude of articles that are the basis of obtaining the various materials used in dentistry that offer good mechanical properties and biocompatibility with dental tissue. Szczesio-Wlodarczyk et al. [26] concluded that a UDMA/Bis-GMA/DMATEG ratio of 40/40/20 wt.% offers the best combination for flexural strength, modulus of elasticity, hardness, diametral compression and water sorption.

In addition to the classic mixture of monomers, a system of monomers based on PMMA (40%) and MMA (25%) was also used. PMMA has been used in dentistry for many years as a base material for prostheses [27] together with MMA [28], which is also used in the adhesion processes of restorative materials.

Moreover, previous research has reported the use of several fillers to reinforce polymer structures, such as glass powders and graphene [29,30]. Since the loading of polymer resins with graphene influences the formation of aggregates that stiffen the mixture, we chose to use the lowest value found in the literature, of 0.5% [31], and which would still offer improvements in the mechanical parameters.

The samples manufactured in the present study, using the polymer mixture, were subjected to mechanical and sorption tests to know the behavior of their structure under certain forces or environments. If the structure of the final hardened mixture is not compact, fractures or defects may appear in the final products [32], which implicitly provide a low mechanical performance. 

In the present study, the best mechanical results were obtained for samples R3, R3 + P, R4 and R4 + P when compared to R1, R1 + P, R2 and R2 + P. The difference between these two sample groups is represented by the precursors used in the polymer matrix. The linear polymer chains of polymethyl methacrylate in the reticulated matrix of Bis-GMA can increase the breaking strength of the mixtures.

Comparing the results obtained for all three mechanical tests, sample R4 + P obtained the highest values for mechanical resistance both in compression resistance, resistance in diametral compression and flexural strength. It has 5% graphene in its composition, which is known to improve the properties of polymers significantly, especially mechanical properties.

Another factor that influences the material characteristics is the exposure to the wet oral environment of the polymer network that can favor water absorption, which initiates the hydrolytic degradation of the ester bonds, creating cracks that yield more easily under the action of mechanical forces [33]. The present report evaluated the flexural strength. Some variables can have an influence on material behavior, such as curing type [34], fatigue [35], and roughness [36]; thus, future studies are needed for a more complete understanding of Polymer mixtures for self-limited dental burs.

Comparing the samples with PMMA (R3, R4 with and without powder) with pure PMMA from the literature studies [37], their resistances increase with the addition of dilution monomer and powders. For samples with Bis-GMA/TEGDMA/UDMA, when compared to other studies with the same ratio (40/40/20) [38], the flexural strengths are almost identical, as the incorporation of the powder is useful in increasing the strength parameters.

It is observed that the abrasion resistance is lower for the samples with the inorganic filler, because the polymer matrix is softer and less resistant to wear than the inorganic filler [39]. The use of smaller particles creates the possibility of minimizing the space between the particles, obtaining a dense composition with good mechanical resistance and good plasticity, and being able to fulfill the characteristics of polymer self-limited burs.

The limitations of this study depend on the lack of documentation regarding the composition and physical–mechanical characterization of the experimental polymer burs. There is no database regarding their inclusion within certain limits, the comparison being made only with mixtures of dental polymers.

## 5. Conclusions

This study assessed the main mechanical properties, water sorption and microscopic structure of Bis-GMA/TEGDMA/UDMA and Bis-GMA/PMMA/MMA samples loaded or not with 5% glass and 0.5% Gr-Ag, focusing on the practical implications of these mixtures in obtaining polymer burs with practical applications. The results revealed a significantly improved mechanical behavior of the PMMA samples enriched with Gr-Ag in terms of compression, flexure, and water sorption behavior. From the mechanical evaluation, we could conclude that the Bis-GMA/PMMA/MMA mixtures fit the mechanical characteristics supported by polymer burs, following future studies regarding their use on affected dentin.

Unfortunately, there are no data in the specialized literature regarding the composition and types of polymers used in obtaining such smart polymer burs; this article is based only on obtaining information in comparison with dental materials. The following studies of the present one will deepen in obtaining and analyzing the burs on the extracted teeth.

## Figures and Tables

**Table 1:** Polymer mixture compositions: Bis-GMA: Bis-phenol A diglycidyl ether dimethacrylate; UDMA: Urethane-dimethacrylate; TEGDMA: triethylene glycol dimethacrylate; MMA: Methyl-methacrylate; PMMA: Poly (methyl methacrylate); CQ: Camphorquinone; DMAEM: 2-(Dimethylamino) ethyl methacrylate.

Recipe	Organic Phase	Inorganic Phase	Initiation System
R1	Bis-GMAimp 40%; UDMA 20%; TEGDMA 40%		1% Amina photo (DMAEM), 0.5% CQ
R1 + P	Bis-GMAimp 40%; UDMA 20%; TEGDMA 40%	Glass containing 5% BaF_2_; Graphene containing 0.5% Ag	
R2	Bis-GMA 40% (2018); UDMA 20%; TEGDMA 40%		
R2 + P	Bis-GMA 40% (2018); UDMA 20%; TEGDMA 40%	Glass containing 5% BaF_2_; Graphene containing 0.5% Ag	
R3	Bis-GMA imp 10%; UDMA 25%; PMMA 40%; MMA 25%		
R3 + P	Bis-GMAimp 10%; UDMA 25%; PMMA 40%; MMA 25%	Glass containing 5% BaF_2_; Graphene containing 0.5% Ag	
R4	Bis-GMA 10% (2018); UDMA 25%; PMMA 40%; MMA 25%		
R4 + P	Bis-GMA 10% (2018); UDMA 25%; PMMA 40%; MMA 25%	Glass containing 5% BaF_2_; Graphene containing 0.5% Ag	
Recipe	Organic phase	Anorganic phase	

**Table 2:** *p* values depending of immersion time.

Samples	*p* Values for			
	Sorption in Artificial Saliva	Sorption in SBF	Solubility in Artificial Saliva	Solubility in SBF
R1	2.50464 × 10^−7^	0.02205	0.00172	7.59885 × 10^−4^
R1 + P	0.00512	0.10036	1.3627 × 10^−4^	8.28607 × 10^−4^
R2	0.01588	0.06988	1.09785 × 10^−5^	5.9272 × 10^−7^
R2 + P	0.45673	0.22578	1.22767 × 10^−5^	0.00546
R3	0.15128	0.00463	0.00525	0.00144
R3 + P	0.00623	0.01337	0.96261	0.30703
R4	0.21407	8.94517 × 10^−4^	4.9139 × 10^−4^	1.21355 × 10^−6^
R4 + P	0.00125	0.02541	0.68245	0.15428

**Table 3:** Mean height (mm ± SD) and weight (g ± SD) before and after abrasion.

Samples	Height		Weight	
	Baseline	After Cycles	Baseline	After Cycles
R1	5.99 ± 0.21	5.38 ± 0.18	0.0809 ± 0.002	0.0779 ± 0.001
R1 + P	5.95 ± 0.08	5.54 ± 0.14	0.0795 ± 0.001	0.0766 ± 0.002
R2	6.01 ± 0.10	5.68 ± 0.09	0.0794 ± 0.002	0.0735 ± 0.001
R2 + P	6.21 ± 0.13	6.08 ± 0.11	0.0825 ± 0.003	0.0801 ± 0.003
R3	6.11 ± 0.10	5.77 ± 0.08	0.0804 ± 0.001	0.0789 ± 0.001
R3 + P	6.31 ± 0.12	6.05 ± 0.13	0.0802 ± 0.002	0.0793 ± 0.002
R4	6.15 ± 0.09	5.99 ± 0.07	0.0840 ± 0.001	0.0831 ± 0.001
R4 + P	6.22 ± 0.06	6.06 ± 0.05	0.0820 ± 0.002	0.0814 ± 0.003

## Data Availability

The data presented in this study are available on request from the authors.
